# Structural validation of two person-centred practice inventories PCPI-S and PCPI-C - French version

**DOI:** 10.1186/s12913-024-11432-y

**Published:** 2024-09-18

**Authors:** Cedric Mabire, Marie Piccot-Crezollet, Vaibhav Tyagi, Brendan McCormack, Joanie Pellet

**Affiliations:** 1 Institute of Higher Education and Research in Healthcare (IUFRS), Lausanne University Hospital, University of Lausanne, Lausanne, Switzerland; 2 Geneva Institution for Home Care and Assistance (IMAD), Geneva, Switzerland; 3 Sydney Nursing School, Faculty of Medicine and Health, University of Sydney, Camperdown, NSW Australia

**Keywords:** Person-centred care, Person-centred practice inventory, Acute care, Confirmatory factor analysis, Psychometric analysis

## Abstract

**Background:**

The shift towards person-centred care has become integral in achieving high-quality healthcare, focusing on individual patient needs, preferences, and values. However, existing instruments for measuring person-centred practice often lack theoretical underpinnings and comprehensive assessment. The Person-centred Practice Inventory – Staff (PCPI-S) and the Person-centred Practice Inventory – Care (PCPI-C) were developed in English to measure clinicians’ and patients’ experience of person-centred practice. The aim of this study was to investigate the psychometric properties of the French version of the PCPI-S and PCPI-C.

**Methods:**

A multi-centred cross-sectional study was conducted in six hospitals in French-speaking Switzerland. Construct validity of the PCPI-S and the PCPI-C was evaluated by using confirmatory factor analysis and McDonald’s Omega coefficient was used to determine the internal consistency.

**Results:**

A sample of 558 healthcare professionals and 510 patients participated in the surveys. Psychometric analyses revealed positive item scores and acceptable factor loadings, demonstrating the meaningful contribution of each item to the measurement model. The Omega coefficient indicated acceptable to excellent internal consistency for the constructs. Model fit statistics demonstrated good model fit for the PCPI-S and PCPI-C.

**Conclusions:**

The findings support the construct validity and internal consistency of the PCPI-S and PCPI-C in assessing person-centred practice among healthcare professionals and patients in French-speaking Switzerland. This validation offers valuable tools for evaluating person-centred care in hospital settings.

**Supplementary Information:**

The online version contains supplementary material available at 10.1186/s12913-024-11432-y.

## Background

Person-centred care is an approach to healthcare that prioritises the individual needs, preferences, and values of the patient [1]. This approach recognises the fundamental role of patients as active participants in their own care, emphasizes the genuine relationship between patients and health professionals and acknowledges the context in which the care is delivered [2]. The shift towards person-centred care has gained momentum over the past few decades and become essential for achieving high-quality healthcare [3]. Person-centred care is of particular interest to politicians, researchers, and clinicians, as it is associated with improved clinical outcomes [4, 5], patient satisfaction [4, 6, 7], work environment factors [8] and economic outcomes [9, 10]. Person-centred care has been implemented across various healthcare settings, including primary care, long-term care and acute care facilities [11, 12].

The Person-centred Practice Framework (PCPF) was developed by McCormack and McCance to support healthcare professionals to understand the dimensions of person-centredness and how to implement person-centred care in clinical practice. The PCP Framework comprises five interrelated domains: macro-context, prerequisites, care environment, person‐centred processes, and person‐centred outcomes. The macro-context domain refers to broader societal, cultural, and policy-related factors that influence healthcare practices. The prerequisites domain emphasises the essential organisational and practice-level elements required to support person-centred care. The care environment domain centres on the physical and emotional context in which care is provided. The person-centred processes domain highlights the importance of effective communication, engagement, and collaborative decision-making between patients and healthcare providers, fostering meaningful partnerships in care. Finally, the person-centred outcomes domain focuses on the positive impacts of person-centred care on patients [1].

Evaluation of person-centred practice is essential for identifying areas for improvement and monitoring its effective implementation within healthcare organisations [13]. Measurement tools can provide a standardised approach to assess the extent to which care aligns with person-centred principles and to support healthcare professionals in enhancing quality-of-care delivery and tailoring services to meet individual needs [14]. However, most of the available instruments measuring person-centred practice lack theoretical underpinnings or fail to assess the various aspects of person-centred care comprehensively [14, 15]. To address the need of demonstrating the value of person-centred care, the PCPF has guided the development of measurement tools. The Person-Centred Practice Inventory – Staff (PCPI-S) developed by Slater et al. and the Person-Centred Practice Inventory – Care (PCPI-C) are aligned with key dimensions of the PCP Framework, including prerequisites, care environment, and person-centred processes [1, 16, 17]. The psychometric properties of the original version of the PCPI-S are acceptable (root mean square error of approximation (RMSEA) = 0.053, comparative fit index (CFI) = 0.951) with reference to the COnsensus-based Standards for the selection of health Measurement INstruments (COSMIN) criteria: CFI > 0.95, RMSEA < 0.06, standardised root mean residual (SRMR) < 0.08 [16, 18]. The PCPI-S was designed for and tested with health care staff across all healthcare settings [16, 19–24]. The instrument has been developed in English [9] and then translated into Swiss German, German, Austrian, Norwegian, Malaysian, Spanish and Portuguese [19–25]. The psychometric properties of the original version of the PCPI-C have not yet been published. By capturing the perspectives of both healthcare professionals and patients, the PCPI-S and the PCPI-C provide a comprehensive assessment of person-centred care [16]. Validation efforts are required to determine whether the PCPI-S and the PCPI-C translated into French provide valid measures of person-centred practice [16].

### Aim

The aim of this study was to evaluate the construct validity and internal consistency of the PCPI-S and the PCPI-C among health care staff and patients in the French-speaking part of Switzerland.

## Method

### Design and setting

This multi-centred cross-sectional study was conducted between March and August 2022. We invited Chief Nursing Officers (CNOs) of major public hospitals in the French-speaking part of Switzerland to participate. Out of those contacted, six hospitals agreed to take part in the study. Following this initial outreach, the project was introduced to the departments selected by the CNO. Subsequently, the unit participation was determined by the management teams. Notably, there were no specific criteria for the selection of units, as the PCPI-S and PCPI-C were intended for use across various healthcare settings and by professionals of different disciplines. Participating study sites included medical and surgical units, obstetrics/gynaecology/maternity, oncology, rehabilitation and geriatrics, neurology, outpatient care, and psychiatry.

### Person-centred practice inventory

The PCPI-S and the PCPI-C were translated into French prior to this study by using principles of good practice for the translation and cultural adaptation of patient reported outcomes measures [26]. Two nurses with a master’s degree, independently translated the PCPI-S and PCPI-C into French and then confer to reach consensus on the provisional forward translation. Then, two other back translators were blind to the source language scales. Finally, a consensus was reach with the translation team.

The PCPI-S consists of 17 dimensions with 59 items about the three domains of the theoretical framework: prerequisites, care environment, and person-centred process. The prerequisites include five constructs: being professionally competent (Q1-Q3), developing interpersonal skills (Q4-Q7), showing commitment to work (Q8-Q12), knowing oneself (Q13-Q15), and being able to clearly demonstrate one’s beliefs and values (Q16-Q18) [27]. The care environment comprises seven constructs: appropriate skill mix (Q19-Q21), shared decision-making system (Q22-Q25), effective relationships between team members (Q26-Q28), power sharing (Q29-Q32), potential for innovation and risk-taking (Q33-Q35), physical environment (Q36-Q38), and supportive organisational system (Q39-Q43) [27]. The person-centred processes have five constructs: working with the patient’s beliefs and values (Q44-Q47), shared decision-making (Q48-Q50), authentic engagement in the relationship (Q51-Q53), being present with caring (Q54-Q56), and working holistically with the whole person (Q57-Q59).

Items are scored on a 5-point Likert scale ranging from 1 (“strongly disagree”) to 5 (“strongly agree”). The score for each construct is obtained by averaging the total items in the construct. The total score is obtained by averaging the scores of the constructs. Pearson’s correlation coefficient is used to calculate the correlations between the three main domains of the PCPI-S (prerequisites, care environment, and person-centred process).

The PCPI-C comprises 18 items aimed at evaluating patients’ agreement levels with statements regarding the person-centred process dimensions described in the PCPF. The PCPI-C comprises five constructs: working with the person’s beliefs and values (Q1-14-7-6), sharing decision-making (Q3-17-20-10), engaging authentically (Q12-18-9), being sympathetically present (Q16-5-2), and working holistically (Q 15-8-4-19). The PCPI-C uses a 5-point Likert scale ranging from 1 (“strongly disagree”) to 5 (“strongly agree”). The score for each construct is obtained by averaging the scores of the items in the construct. The total score is obtained by averaging the scores of the constructs.

The following characteristics were collected from health care staff: gender, age, profession, level of training, additional training, years of experience, care unit, activity rate, and years of experience in the current unit. Patients characteristics were retrieved from health electronic records and included gender, age, length of hospital stay at the time of completing the PCPI-C and whether patients were in single or shared room as it could influence the perception of care environment.

### Participants

All health care staff members from participating units who were directly involved in patient care were invited to participate in completing the PCPI-S. A sample of patient participants was recruited on a voluntary basis from the participating units. Inclusion criteria for patients included being 18 years or older, proficient in reading and understanding French, and deemed cognitively capable by the healthcare team to complete the PCPI-C. The target sample size was 600 healthcare staff members and 200 patients to meet the criteria defined by the COSMIN [18].

### Data collection

An email containing the URL to access the online PCPI-S was sent to healthcare staff members within the participating units. A data collection day was organized at each participating unit in the six hospitals. During this day, eligible patients were identified by the healthcare team. The study’s purpose and questionnaire were orally explained to the participants by the researcher. For participants capable of completing it independently, the PCPI-C paper questionnaire was provided and collected after completion at the end of the day. For patients who were unable to complete the questionnaire due to visual or motor impairments, the researcher either assisted in reading the questionnaire or provided physical support. The researcher paid careful attention to reading the questionnaire faithfully and avoiding influencing the participants’ responses.

### Statistical analysis

Descriptive statistical analyses of the instruments and participants’ characteristics were performed by calculating mean and standard deviation.

For assessing psychometric properties, confirmatory factor analysis (CFA) was performed based on the structure of the PCPF theoretical framework. The parameters of the structural equation model were estimated by using the maximum likelihood ratio method. Missing data were left in the analyses and the maximum likelihood with missing (MLMV) model was used in Structural Equation Modelling. The internal consistency of the instruments was determined by using the McDonald’s Omega coefficient. The Omega coefficient can be judged as acceptable at over 0.70. Model fits were assessed using three fit indices and their goodness of fit criteria: root mean square error of approximation (RMSEA) (< 0.08), comparative fit index (CFI) (> 0.90), and standardized root mean square residuals (SRMR) (< 0.08). At least one of these criteria should be met to support the construct validity [28]; if the non-centrality index, RMSEA, is > 90%; and if its parsimony index, the Akaike information criterion, is lowest [29]. Analyses were performed by using Stata/IC software 17 [30].

### Ethical considerations

The study was submitted and approved by the ethics committee of the canton of Vaud (CER-VD 2020 − 01562). All participants were informed about the study and gave consent to participate.

## Results

### Participant characteristics

A total sample of 558 healthcare staff members completed the PCPI-S. They were predominantly women (85%) and worked as nurses (62%). Most staff members worked in medical (33%) and surgical wards (15%). Patient participants (*n* = 510) were 70 years old on average and women accounted for half of the sample (51%). The mean length of stay when completing the PCPI-C was 11 days. Patients were mostly hospitalised in medical (36%) and surgical wards (27%) (Table 1).

**Table 1:** Characteristics of the participants

Characteristics	N
**Health care staff**	*n* = 558
Gender	
Women, n (%)	429 (84.6)
Age, M (SD)	39.4 (11.5)
Profession, n (%)	
Nurse	333 (62.4)
Physiotherapist/Occupational therapist	46 (8.2)
Midwife	38 (7.1)
Nurse assistant	33 (6.2)
Other	84 (15)
Years of experience, M (SD)	14.7 (11.1)
Years in current unit, M (SD)	7.6 (8.2)
Department, n (%)	
Medicine	167 (33)
Surgery	78 (15.4)
Readaptation and geriatrics	66 (13)
Obstetrics/gynaecology/maternity	41 (8.1)
Outpatient care	23 (4.5)
Oncology	10 (2)
Other	121 (23.9)
**Patients**	*n* = 510
Gender	
Women, n (%)	260 (51.2)
Age, M (SD)	69.6 (69.6)
Department, n (%)	
Medicine	181 (35.8)
Surgery	135 (26.7)
Readaptation and geriatrics	93 (18.4)
Outpatient care	25 (5)
Geriatric	15 (3)
Obstetrics/gynaecology/maternity	14 (2.8)
Other	42 (8.2)
Hospitalisation days, M (SD)	11 (15)
Private room, n (%)	113 (23.8)
Shared room, n (%)	362 (76.2)

Note: M = mean; SD = standard deviation

### Psychometric analysis

All items of the PCPI-S and PCPI-C received positive scores, with mean scores ranging from 2.49 to 4.54. For the patient sample, there were 4 missing responses (0.8%) for questions 1 and 3, to 14 missing responses (2.8%) for questions 19 and 20. For the caregiver sample, there were 1 missing responses (0.2%) for questions 2 to 6, up to 68 missing responses (15%) for questions 44 to 59. Pearson’s correlation coefficient indicates statistically significant positive correlations between the three main domains of the PCPI-S: prerequisites and care environment (*r* = 0.57, *p* < 0.01), prerequisites and person-centred process (*r* = 0.72, *p* < 0.01), and care environment and person-centred process (*r* = 0.49, *p* < 0.01). Factor loadings ranged from 0.35 to 0.89, with the majority exceeding 0.5. Notably, all factor loadings were statistically significant (standard error < 0.9; *p* < 0.01) and made meaningful contributions to the measurement model. As a result, these items were retained in the analysis [31]. Detailed factor loadings are presented in additional files 1 and 2.

In the case of the PCPI-S scale, the Omega coefficients for each domain were deemed acceptable, ranging from 0.87 for the Prerequisites factor to 0.93 for Person-centred processes. The specific Omega coefficients for each factor can be found in additional files 1 and 2. Regarding the PCPI-C scale, the Omega coefficients for each construct were also found to be acceptable, ranging from 0.64 for the Engaging Authenticity factor to 0.74 for Patient Beliefs and Values. The Omega coefficients for each factor are detailed in additional files 1 and 2. Furthermore, the Omega coefficient for the person-centred processes domain was outstanding, scoring at 0.92.

The model fit statistics of the three constructs indicated a good model fit, with a RMSEA close to 0.06, a 90% higher bracket below 0.09, a CFI of 0.90 or higher, and an SRMR less than 0.08. The detailed scores are set out in Table 2.

**Table 2:** Fit statistics for measurement models of the PCPI-S and PCPI-C

Model	c^2^ (df; *p* value)	CFI	RMSEA	90% CI RMSEA	SRMR
PCPI-S					
Prerequisites	354.6 (125; 0.01)	0.900	0.064	0.056; 0.072	-
Care environment	876.9 (254; 0.01)	0.849	0.078	0.072; 0.084	0.079
Person-centred processes	322.7 (94; 0.01)	0.93	0.08	0.07; 0.089	0.000
PCPI-C					
Person-centred processes	437.7 (124; 0.01)	0.915	0.071	0.063; 0.078	-

Note: df = degree of freedom; CI = confidence interval

## Discussion

The results of the psychometric analysis of the PCPI-S demonstrate good construct validity and internal consistency, thereby confirming the underlying principles of the theoretical PCP Framework. The model fit statistics consistently indicate a good fit for the three constructs within the PCPI-S. The PCPI-C demonstrates a reasonable to acceptable fit, indicating that while the model is not perfect, it is sufficiently robust for practical applications.

Examining the psychometric properties across different linguistic versions of the PCPI-S provides valuable insights into the instrument’s consistency and internal consistency across diverse cultural and linguistic contexts. In the present study, the Omega coefficient values for the PCPI-S and PCPI-C were consistently above 0.70, indicating robust internal consistency. The results for the PCPI-S are in line with previous research conducted in Swiss German, Austrian, Norwegian, Malaysian and Portuguese studies, which reported high Cronbach’s alpha scores (a > 0.70) [19–23, 25]. These findings confirm the instrument’s strong internal consistency when measuring person-centred care constructs.

Regarding the RMSEA values, the PCPI-S versions in Swiss German, German, Norwegian, and Malaysian studies consistently indicated a good model fit, with RMSEA values ranging from 0.041 to 0.078 [19–22]. All these values were close to 0.06, indicating a good model fit. In the PCPI-S French version, RMSEA values ranged from 0.000 to 0.078. Although the RMSEA for prerequisites and the care environment was slightly higher in the French version than in the Swiss German and Norwegian versions, the RMSEA for person-centred processes was notably lower, suggesting an good fit for this construct in the present study.

The CFI values for the PCPI-S were generally above 0.90 across different linguistic versions, supporting the instrument’s construct validity and internal consistency. In the present study, the CFI values ranged from 0.85 for the care environment to 1.00 for person-centred processes, indicating an excellent fit for this construct. However, the slightly lower CFI for the care environment was consistent with findings in other studies.

The variations observed in different studies across languages may be attributed to linguistic nuances, cultural differences, or contextual factors specific to each linguistic group. These differences highlight the importance of adapting the instruments to the cultural and linguistic context in which they are used, emphasizing the ongoing need for validation and adaptation efforts. The findings from translations into French, Swiss German, German, Norwegian, and Malaysian languages collectively underscore the robustness and adaptability of the PCPI as a tool for assessing person-centred practice in diverse cultural contexts. The consistently high Cronbach’s alpha scores, meaningful factor loadings, and favourable GFIs in these translations suggest that the PCPI maintains its internal consistency and construct validity when applied in different linguistic and cultural settings.

### Implications for clinical practice and future research

The PCPI has demonstrated strong internal consistency and good model fit across different linguistic versions. While the French-translated PCPI-S shows promising construct validity, its length may pose a challenge for widespread clinical adoption. Considering the time constraints frequently encountered in healthcare settings, there is a need for future research to design a shorter yet psychometrically robust version of the scale. This would enable quicker and more efficient assessments of patient-centred care without compromising measurement quality.

The availability of instruments aligned with a theoretical person-centred framework provides healthcare staff with a standardised measure to evaluate the degree of alignment with person-centered principles in care delivery. Consistent use of the PCPI-S and PCPI-C enable healthcare staff to collectively identify areas that require improvement, thereby fostering a continuous quality improvement process. Furthermore, insights gained from the PCPI-S and PCPI-C could inform the development of training programs aimed at enhancing person-centered care competencies among healthcare professionals.

### Strengths and limitations

The large participation of both healthcare staff members and patient from six hospital and multiple clinical settings enhance generalisability of the results and confidence in the findings. Nonetheless, certain limitations should be acknowledged. The sample predominantly comprising nurses, and the relative homogeneity in participants’ responses could suggest a limited familiarity with the person-centred principles and the PCPF among both professionals and patients. While this study used CFA for psychometric analysis, further psychometric validation of the PCPI-S in French should include additional analyses such as a test-retest procedure and concurrent validity assessment. Finally, as professionals and patients participated on a voluntary basis, we cannot exclude a potential selection and desirability bias.

## Conclusions

The psychometric analysis conducted in this study indicates high construct validity and internal consistency for the French translation of both the PCPI-S and the PCPI-C. The results presented in this article will enable international comparative studies and support the further development of person-centred care in French-speaking clinical settings.

## Electronic supplementary material

Below is the link to the electronic supplementary material.

Supplementary Material 1

## Data Availability

The datasets used and analysed during the current study are available on www.zenodo.org. DOI: 10.5281/zenodo.10849449.

## Abbreviations

PCPI-S: Person-centred Practice Inventory – Staff
PCPI-C: Person-centred Practice Inventory – Care
CFA: Confirmatory factor analysis
PCPF: Person-centred Practice Framework
COSMIN: Consensus-based Standards for the selection of health Measurement Instruments
RMSEA: Root mean square error of approximation
SRMR: Standardised root mean residual
CFI: Comparative fit index
GFI: Goodness-of-fit index
CI: Confidence interval
