# Supplementary material for: Structural validation of two person-centred practice inventories PCPI-S and PCPI-C - French version
Source: BMC Health Serv Res. 2024 Sep 18;24:1092. doi: 10.1186/s12913-024-11432-y (PMC11412049; doi:10.1186/s12913-024-11432-y)
Supplement: Supplementary file 1 — Supplementary Material 1 [file 12913_2024_11432_MOESM1_ESM.docx]

**Additional file 1.** Mean, measures of distribution and factor loadings of PCPI-S items

| **Construct scores and items** | **Mean** | **SD** | **Factor loading** | **SE** |
| --- | --- | --- | --- | --- |
| **Prerequisites** (Omega’s alpha) | | | | 0.87 |
| *Professionally competent* (Omega’s alpha) | | | | 0.48 |
| Q1. I have the necessary skills to negotiate care options. | 4.06 | 0.67 | 0.43 | 0.05 |
| Q2. When I provide care I pay attention to more than the immediate physical task. | 4.44 | 0.70 | 0.36 | 0.05 |
| Q3. I actively seek opportunities to extend my professional competence. | 4.21 | 0.72 | 0.58 | 0.04 |
| *Developed interpersonal skills* (Omega’s alpha) | | | | 0.75 |
| Q4. I ensure I hear and acknowledge others’ perspectives. | 4.42 | 0.55 | 0.70 | 0.03 |
| Q5. In my communication I demonstrate respect for others. | 4.54 | 0.57 | 0.63 | 0.04 |
| Q6. I use different communication techniques to find mutually agreed solutions. | 4.15 | 0.70 | 0.67 | 0.03 |
| Q7. I pay attention to how my non-verbal cues impact on my engagement with others. | 4.09 | 0.72 | 0.63 | 0.04 |
| *Being committed to the job* (Omega’s alpha) | | | | 0.73 |
| Q8. I strive to deliver high quality care to people. | 4.58 | 0.56 | 0.50 | 0.04 |
| Q9. I seek opportunities to get to know the person and their family in order to provide holistic care. | 4.10 | 0.81 | 0.58 | 0.04 |
| Q10. I go out of my way to spend time with people receiving care. | 4.11 | 0.73 | 0.62 | 0.04 |
| Q11. I strive to deliver high quality care that is informed by evidence. | 4.18 | 0.68 | 0.57 | 0.04 |
| Q12. I continuously look for opportunities to improve the care experiences. | 4.16 | 0.69 | 0.68 | 0.03 |
| *Knowing self* (Omega’s alpha) | | | | 0.78 |
| Q13. I take time to explore why I react as I do in certain situations. | 3.98 | 0.82 | 0.74 | 0.03 |
| Q14. I use reflection to check out if my actions are consistent with my ways of being. | 4.19 | 0.68 | 0.75 | 0.03 |
| Q15. I pay attention to how my life experiences influence my practice. | 4.08 | 0.78 | 0.73 | 0.03 |
| *Clarity of beliefs and values* (Omega’s alpha) | | | | 0.67 |
| Q16. I actively seek feedback from others about my practice. | 3.69 | 0.94 | 0.51 | 0.05 |
| Q17. I challenge colleagues when their practice is inconsistent with our team’s shared values and beliefs. | 3.42 | 0.95 | 0.65 | 0.04 |
| Q18. I support colleagues to develop their practice to reflect the team’s shared values and beliefs. | 3.99 | 0.78 | 0.75 | 0.04 |
| **The care environment** (Omega’s alpha) | | | | 0.90 |
| *Skill mix* (Omega’s alpha) | | | | 0.41 |
| Q19. I recognise when there is a deficit in knowledge and skills in the team and its impact on care delivery. | 3.97 | 0.84 | 0.35 | 0.07 |
| Q20. I am able to make the case when skill mix falls below acceptable levels. | 3.70 | 0.91 | 0.50 | 0.06 |
| Q21. I value the input from all team members and their contributions to care. | 4.34 | 0.64 | 0.43 | 0.07 |
| *Shared decision-making systems* (Omega’s alpha) | | | | 0.76 |
| Q22. I actively participate in team meetings to inform my decision-making. | 3.97 | 0.92 | 0.58 | 0.04 |
| Q23. I participate in organisation-wide decision-making forums that impact on practice. | 2.89 | 1.27 | 0.76 | 0.03 |
| Q24. I am able to access opportunities to actively participate in influencing decisions in my directorate/division. | 3.37 | 1.09 | 0.76 | 0.03 |
| Q25. My opinion is sought in clinical decision-making forums (e.g. ward rounds, case conferences, discharge planning). | 3.60 | 1.08 | 0.55 | 0.04 |
| *Effective staff relationships* (Omega’s alpha) | | | | 0.83 |
| Q26. I work in a team that values my contribution to person-centred care. | 3.83 | 0.90 | 0.89 | 0.02 |
| Q27. I work in a team that encourages everyone’s contribution to person-centred care. | 3.86 | 0.88 | 0.88 | 0.02 |
| Q28. My colleagues positively role model the development of effective relationships. | 3.66 | 0.84 | 0.58 | 0.04 |
| *Power sharing* (Omega’s alpha) | | | | 0.76 |
| Q29. The contribution of colleagues is recognised and acknowledged. | 3.69 | 0.93 | 0.60 | 0.03 |
| Q30. I actively contribute to the development of shared goals. | 3.81 | 0.83 | 0.51 | 0.04 |
| Q31. The leader facilitates participation. | 3.87 | 1.03 | 0.70 | 0.03 |
| Q32. I am encouraged and supported to lead developments in practice. |  |  | 0.84 | 0.02 |
| *Potential for innovation and risk taking* (Omega’s alpha) | | | | 0.60 |
| Q33. I am supported to do things differently to improve my practice. | 3.55 | 0.98 | 0.84 | 0.03 |
| Q34. I am able to balance the use of evidence with taking risks. | 3.60 | 0.84 | 0.41 | 0.05 |
| Q35. I am committed to enhancing care by challenging practice. | 4.23 | 0.64 | 0.40 | 0.04 |
| *The physical environment* (Omega’s alpha) | | | | 0.69 |
| Q36. I pay attention to the impact of the physical environment on people’s dignity. | 4.40 | 0.62 | 0.57 | 0.05 |
| Q37. I challenge others to consider how different elements of the physical environment impact on person-centredness (e.g. noise, light, heat etc). | 3.92 | 0.80 | 0.71 | 0.04 |
| Q38. I seek out creative ways of improving the physical environment. | 3.51 | 0.94 | 0.67 | 0.04 |
| *Supportive organisational systems* (Omega’s alpha) | | | | 0.84 |
| Q39. In my team we take time to celebrate our achievements. | 2.84 | 1.07 | 0.59 | 0.04 |
| Q40. My organisation recognises and rewards success. | 2.49 | 1.06 | 0.73 | 0.03 |
| Q41. I am recognised for the contribution that I make to people having a good experience of care. | 3.21 | 1.01 | 0.68 | 0.03 |
| Q42. I am supported to express concerns about an aspect of care. | 3.27 | 1.02 | 0.81 | 0.02 |
| Q43. I have the opportunity to discuss my practice and professional development on a regular basis. | 3.20 | 1.05 | 0.77 | 0.03 |
| **Person-centred processes** (Omega’s alpha) | | | | 0.93 |
| *Working with patient beliefs and values* (Omega’s alpha) | | | | 0.82 |
| Q44. I integrate my knowledge of the person into care delivery. | 4.28 | 0.63 | 0.71 | 0.03 |
| Q45. I work with the person within the context of their family and carers. | 4.21 | 0.70 | 0.75 | 0.03 |
| Q46. I seek feedback on how people make sense of their care experience. | 3.80 | 0.84 | 0.70 | 0.03 |
| Q47. I encourage people to discuss what is important to them. | 4.24 | 0.68 | 0.77 | 0.02 |
| *Shared decision-making* (Omega’s alpha) | | | | 0.69 |
| Q48. I include the family in care decisions where appropriate and/or in line with the person’s wishes. | 4.08 | 0.78 | 0.65 | 0.04 |
| Q49. I work with the person to set health goals for their future. | 3.89 | 0.83 | 0.70 | 0.03 |
| Q50. I enable people receiving care to seek information about their care from other healthcare professionals. | 3.90 | 0.79 | 0.60 | 0.04 |
| *Engagement* (Omega’s alpha) | | | | 0.73 |
| Q51. I try to understand the person’s perspective. | 4.13 | 0.70 | 0.75 | 0.03 |
| Q52. I seek to resolve issues when my goals for the person differ from theirs perspectives. | 3.95 | 0.71 | 0.66 | 0.03 |
| Q53. I engage people in care processes where appropriate. | 4.26 | 0.64 | 0.64 | 0.03 |
| *Having sympathetic presence* (Omega’s alpha) | | | | 0.72 |
| Q54. I actively listen to people receiving care to identify unmet needs. | 4.38 | 0.63 | 0.70 | 0.03 |
| Q55. I gather additional information to help me support people receiving care. | 4.08 | 0.75 | 0.71 | 0.03 |
| Q56. I ensure my full attention is focused on the person when I am with them. | 4.18 | 0.71 | 0.62 | 0.03 |
| *Providing holistic care* (Omega’s alpha) | | | | 0.85 |
| Q57. I strive to gain a sense of the whole person. | 4.32 | 0.60 | 0.81 | 0.02 |
| Q58. I assess the needs of the person, taking account of all aspects of their lives. | 4.08 | 0.76 | 0.77 | 0.02 |
| Q59. I deliver care that takes account of the whole person. | 4.26 | 0.65 | 0.85 | 0.02 |

*Note:* SD: standard deviation; SE: standard error

**Additional file 2.** Mean, measures of distribution and factor loadings of PCPI-C items

| **Construct scores and items** | **Mean** | **SD** | **Factor loading** | **SE** |
| --- | --- | --- | --- | --- |
| **Person-centred processes (Omega’s alpha)** | | | | 0.92 |
| *Patient beliefs and values* (Omega’s alpha) | | | | 0.74 |
| Q1. Staff try to understand what is important to me | 4.37 | 0.65 | 0.64 | 0.03 |
| Q14. In caring for me, staff use what they know about me as a person | 4.00 | 0.77 | 0.64 | 0.03 |
| Q7. I feel able to give staff feedback about my experience of being cared for | 4.19 | 0.74 | 0.63 | 0.03 |
| Q6. I feel able to say to staff what is important to me | 4.30 | 0.70 | 0.69 | 0.03 |
| *Shared decision-making* (Omega’s alpha) | | | | 0.73 |
| Q3. Staff involve me in making decisions about my care | 3.99 | 0.92 | 0.69 | 0.03 |
| Q17. Staff help me to express my concerns about my treatment and care | 3.97 | 0.85 | 0.65 | 0.03 |
| Q20. Staff help me to set realistic goals | 3.85 | 0.86 | 0.61 | 0.03 |
| Q10. Staff ask me if I have all the information I need | 3.94 | 0.99 | 0.61 | 0.03 |
| *Engaging authenticity* (Omega’s alpha) | | | | 0.64 |
| Q12. When we disagree about my care, staff try to find common ground | 3.89 | 0.79 | 0.47 | 0.04 |
| Q18. Staff listen to me and hear what I have to say about my care | 4.17 | 0.76 | 0.66 | 0.03 |
| Q9. Staff connect with me as a person | 3.98 | 0.89 | 0.66 | 0.03 |
| *Sympathetic presence* (Omega’s alpha) | | | | 0.69 |
| Q16. Staff respond compassionately when I am upset or unhappy | 4.06 | 0.83 | 0.69 | 0.03 |
| Q5. Staff give me their full attention when they are with me | 4.42 | 0.68 | 0.69 | 0.03 |
| Q2. Staff use my personal experiences to build a relationship with me | 3.78 | 0.90 | 0.60 | 0.03 |
| *Holistic care* (Omega’s alpha) | | | | 0.70 |
| Q15. I feel cared for | 4.48 | 0.62 | 0.69 | 0.03 |
| Q8. Staff ask me about my life | 3.44 | 1.06 | 0.52 | 0.04 |
| Q4. Staff consider my home environment in meeting my care needs | 3.81 | 0.95 | 0.58 | 0.03 |
| Q19. Staff understand my family circumstances when caring for me | 3.76 | 0.84 | 0.59 | 0.03 |

*Note:* SD: standard deviation; SE: standard error

**Additional file 3.** Structural Equation Modelling of PCPI-S

**Additional file 4.** Structural Equation Modelling of PCPI-C
